# Cystic Artery Pseudoaneurysm Secondary to Cholecystitis: A Rare Cause of Hemobilia

**DOI:** 10.7759/cureus.39161

**Published:** 2023-05-17

**Authors:** Haidar Khan, Vennis Lourdusamy, Raghav Bansal

**Affiliations:** 1 Internal Medicine, New York City Health + Hospitals/Elmhurst, New York City, USA; 2 Gastroenterology, New York City Health + Hospitals/Elmhurst, New York City, USA

**Keywords:** pseudoaneurysm of cystic artery, gastrointestinal bleeding, cholecystitis, endoscopy ercp, biliary diseases

## Abstract

Cystic artery pseudoaneurysm (CAP) is usually seen as a complication of cholecystectomy. Infrequently, CAP can develop in the setting of cholecystitis and can present as hemobilia when the aneurysm ruptures. Here, we present the case of an 88-year-old male with hemobilia secondary to CAP which was successfully managed by embolization after an initial biliary stent placement.

## Introduction

Cystic artery pseudoaneurysm (CAP) may occur as a consequence of biliary surgery, especially cholecystectomy [1]. Rarely, CAP can occur secondary to infectious or inflammatory processes of the biliary tree. Clinically, CAP becomes symptomatic after rupture, presenting with upper gastrointestinal bleeding, right upper quadrant pain, and jaundice. Unruptured CAP is clinically silent and can be incidentally discovered on imaging.

## Case presentation

**Figure 1:**
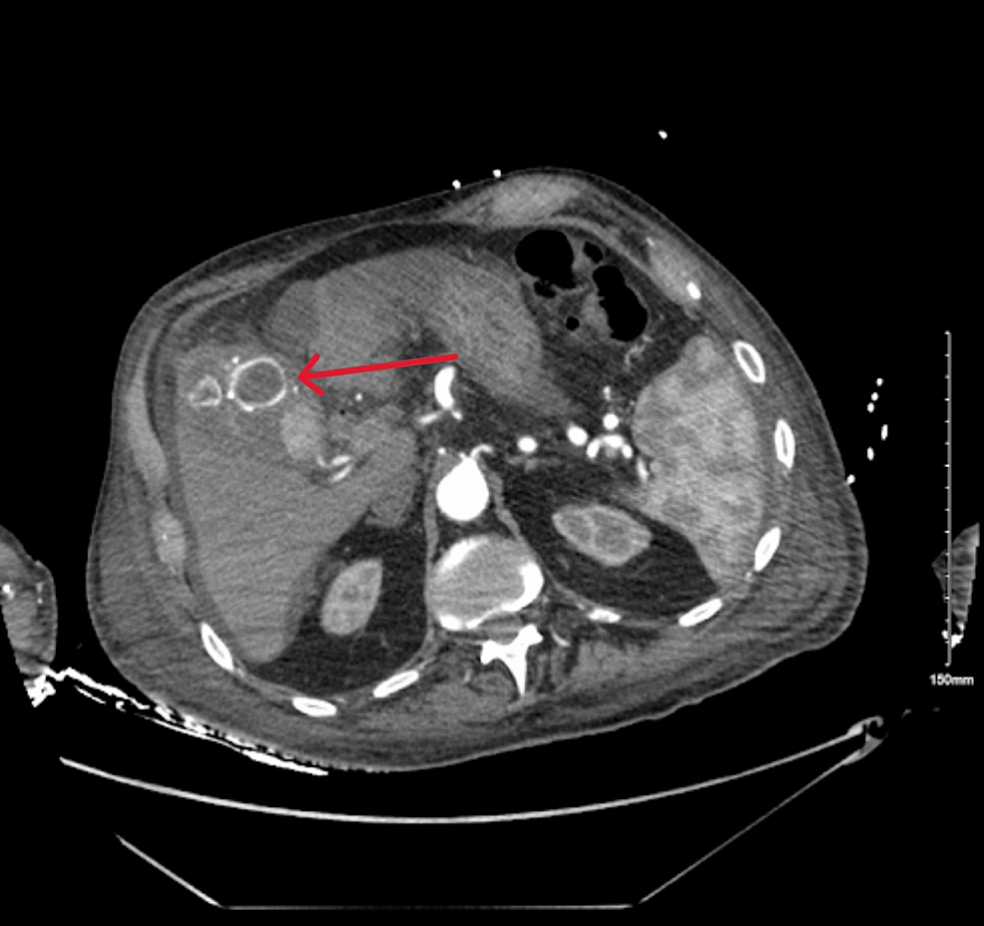
CT of the abdomen with contrast showing evidence of cholelithiasis and intrahepatic dilation.

**Figure 2:**
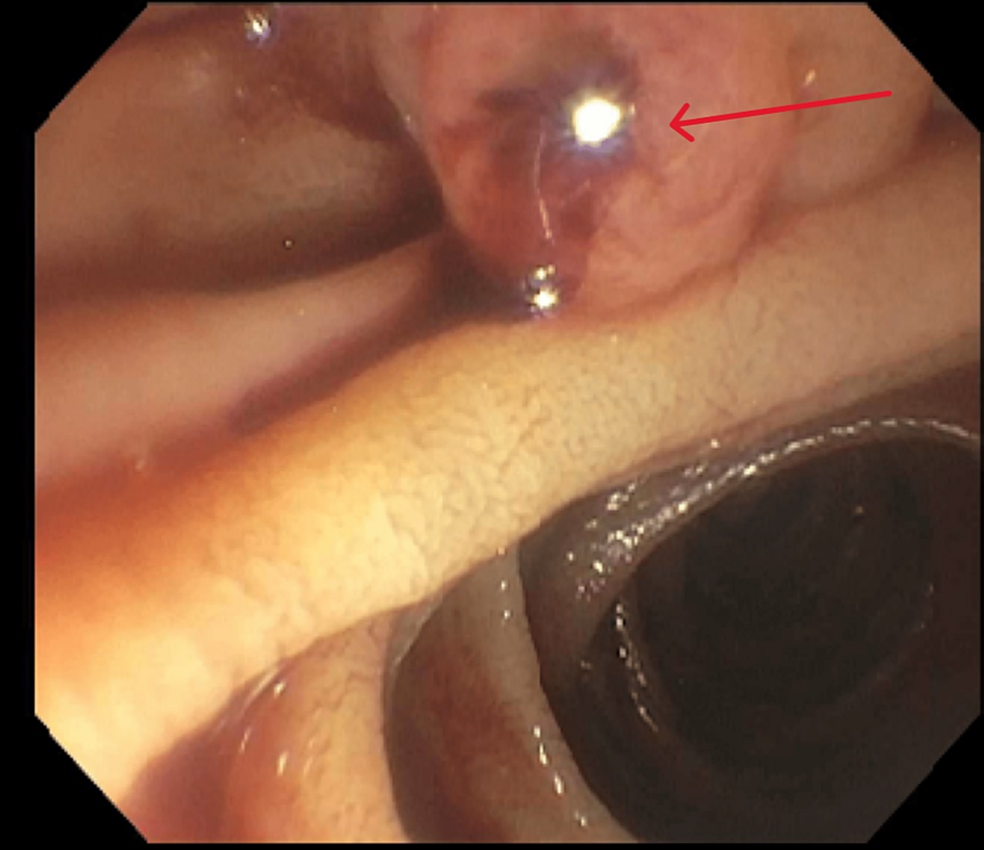
Endoscopic retrograde cholangiopancreatography revealing oozing blood from the ampulla.

**Figure 3:**
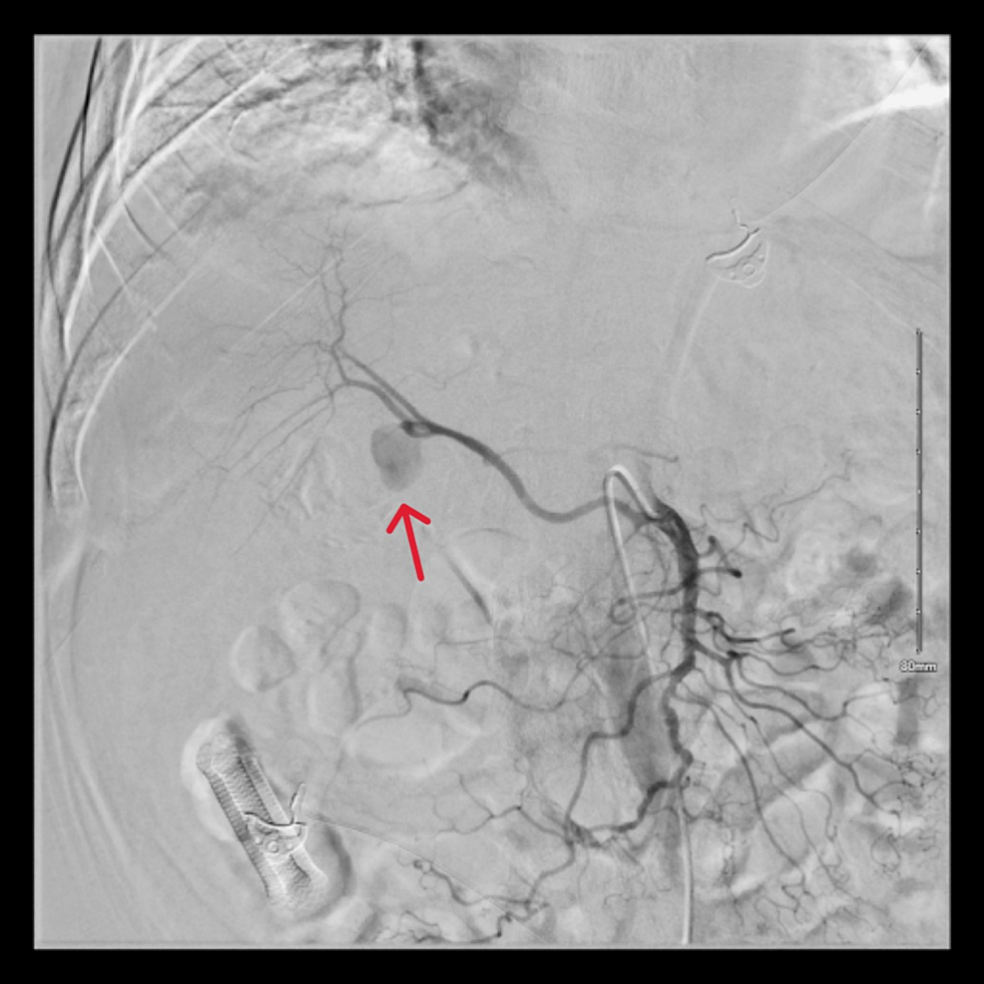
Angiography before embolization showing a cystic artery pseudoaneurysm.

**Figure 4:**
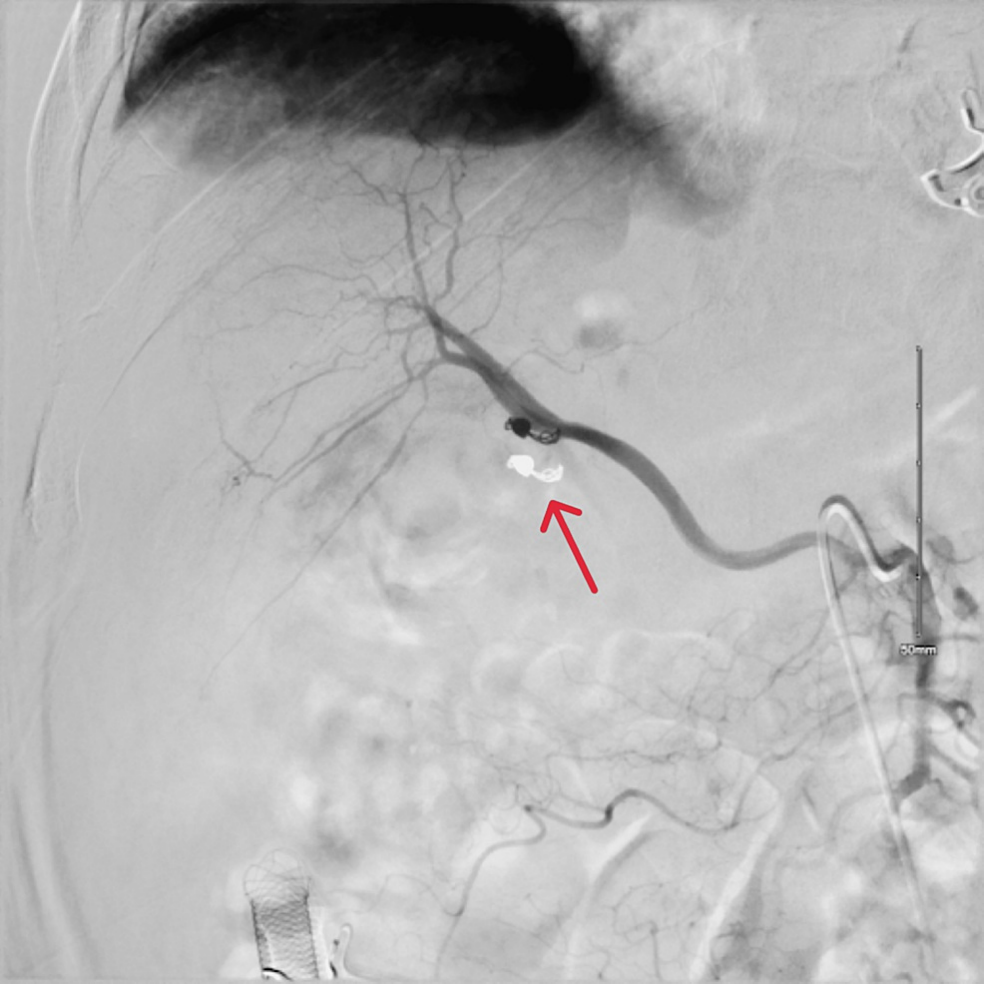
Angiography status after embolization.

An 88-year-old male with a history of hypertension was admitted with fever, chills, and altered mental status. Physical examination revealed jaundice, with right upper quadrant tenderness. Labs showed elevated liver enzymes predominantly in a cholestatic pattern with bilirubinemia (alkaline phosphatase: 728 U/L, aspartate transaminase: 284 U/L, alanine transaminase: 170 U/L, total bilirubin: 3.4 mg/dL, and direct bilirubin: 3.1 mg/dL), leukocytosis, and anemia. CT of the abdomen with contrast showed cholelithiasis, a distended gallbladder, and a dilated common bile duct to 11 mm with intrahepatic dilation without evidence of any pancreatic or ampullary mass (Figure 1). During endoscopic retrograde cholangiopancreatography (ERCP), blood was seen oozing from the ampulla (Figure 2). Stones and blood clots were swept from the duct. A self-expanding metal stent (SEMS) was placed to tamponade the bleed. However, the patient continued to have melena post-procedure requiring a transfusion of 1 unit of packed red blood cells. CT of the abdomen with angiography was performed which showed a large pseudoaneurysm of the cystic artery (Figure 3). The patient then underwent interventional radiology-guided embolization. The inflow from the cystic artery was coiled and the angiogram revealed no residual filling of the cystic artery and the pseudoaneurysm (Figure 4). The patient recovered uneventfully post-embolization and is planned for a cholecystectomy.

## Discussion

Hemobilia usually occurs from an iatrogenic injury involving liver parenchymal or biliary tract instrumentation. Rarely, it can occur from CAP. A literature review performed in 2021 revealed 67 published reports on CAPs since 1991, with the majority of cases secondary to cholecystectomy or cholelithiasis [2]. However, hemobilia secondary to CAP is far rarer, with roughly a dozen cases reported in the last two decades [3].

The proposed mechanisms for pseudoaneurysm formation include an iatrogenic or ischemic injury. It is suggested that the inflammation from cholecystitis may lead to adjacent cystic arterial wall weakening and subsequent pseudoaneurysm formation [2]. CAP can be diagnosed on Doppler sonography, seen as turbulent forward and backward flow within the gallbladder [4]. The most sensitive form of imaging is CT angiography, which often also reveals the cause of CAP [2]. For stable patients, ERCP may be appropriate to visualize hemobilia in the form of oozing blood seen at the ampulla of Vater [2].

The most common treatment modality is endovascular coil embolization of the pseudoaneurysm. For cases identified during cholecystectomy, some literature describes proximal clipping or ligation of the artery as an effective management [5]. Urgent identification and treatment of CAP are critical. There is limited data on the use of SEMS in the tamponade of hemobilia and this remains a promising avenue for further investigation. CAP rupture carries an estimated mortality rate of 21-43% [6].

## Conclusions

Our patient did not have cholecystectomy or prior hepatobiliary instrumentation. Although hepatobiliary malignancies should be suspected, especially in older populations, significant hemobilia requiring transfusion post-ERCP with stent placement should raise suspicion for alternative causes including CAP rupture. Timely CT angiography to guide embolization is crucial. Interventional radiology-guided embolization is the least invasive and effective treatment modality to achieve durable hemostasis. The risk of gangrenous cholecystitis post-embolization is extremely rare, and our patient recovered well post-embolization and is planned for interval cholecystectomy.
